# Advances in organoids for personalized medicine: from technological development to clinical application

**DOI:** 10.3389/fcell.2026.1890385

**Published:** 2026-08-24

**Authors:** Xinkui Zhou, Xingxue Yan, Zhengyang Zhang, Zhilei Zhang, Yixiang Que, Yanfan Wang, Yaodong Zhang, Kuo Lu, Longfei Wang

**Affiliations:** 1 Henan Key Laboratory of Children’s Genetics and Developmental Diseases, Henan International Joint Laboratory of Children’s Infectious Diseases, Henan Clinical Research Center of Childhood Diseases, Children’s Hospital Affiliated to Zhengzhou University, Zhengzhou, Henan, China; 2 Institute of Children’s Health, Henan Academy of Innovations in Medical Science, Zhengzhou, Henan, China; 3 Zhixing Academy, North Henan Medical University, Xinxiang, China

**Keywords:** disease modeling, drug screening, organoids, personalized medicine, precision medicine

## Abstract

Organoids, three-dimensional cell culture models derived from patient tissues or stem cells, have emerged as a cutting-edge technology in personalized medicine, owing to their remarkable ability to closely recapitulate *in vivo* tissue architecture and function. This review provides a comprehensive overview of the technological evolution and construction methodologies of organoids, highlighting their significant applications in oncology, genetic disorders, infectious diseases, and drug screening. This review examines how organoids enable precision medicine by preserving genomic fidelity, predicting drug sensitivity, and creating disease models via gene editing. Despite these advances, organoid technology faces several technical challenges that impede its full clinical translation. Addressing these obstacles is critical for realizing the potential of organoids in individualized therapeutic strategies. This article aims to delineate current progress and future directions in organoid research, furnishing a theoretical foundation and guiding future investigations towards enhancing personalized treatment paradigms.

## 1 Introduction

The emergence of organoid technology marks a transformative milestone in personalized medicine. It offers unprecedented opportunities to recapitulate the complex architecture, cellular heterogeneity, and functional characteristics of human organs *in vitro*. Organoids are three-dimensional (3D) cellular assemblies derived from stem cells or primary tissues. They faithfully mimic the microenvironment and physiological conditions of their tissue of origin, thereby bridging the gap between traditional two-dimensional (2D) cultures and *in vivo* models. Advances in stem cell biology, gene editing, and bioengineering have enabled organoids that retain the genetic and phenotypic features of native tissues. Seminal studies include long-term 3D culture of intestinal stem cells (Sato et al., 2009) and cerebral organoids from iPSCs (Lancaster et al., 2013). Together with subsequent work on Lgr5+ organoids (Barker et al., 2007), these contributions established the framework for current personalized medicine applications.

In oncology, patient-derived organoids (PDOs) have emerged as powerful platforms for drug screening, biomarker discovery, and elucidation of resistance mechanisms. They facilitate the development of precision therapies tailored to individual patients (Abbasian et al., 2025; Vella et al., 2024). Beyond cancer, organoid technology has found broad applications in modeling genetic, infectious, metabolic, and degenerative diseases, offering platforms for mechanistic studies, drug screening, and regenerative medicine (Chu et al., 2026; Chen et al., 2024; Kuhl et al., 2023; Yu, 2021). Furthermore, organoids serve as versatile tools for drug development, enabling high-throughput efficacy testing and toxicity evaluation (Ren et al., 2022; Patel and Amin, 2026).

Building upon these applications, technological innovations have enhanced the fidelity and utility. 3D bioprinting enbales precise spatial organization of multiple cell types (Yu, 2021; Ren et al., 2022), and biomaterial scaffolds provide microenvironments that mimic the extracellular matrix (Zhao et al., 2026; Shao et al., 2025). These approaches overcome traditional limitations, such as vascularization, immune components, and standardized conditions, and improve reproducibility and scalability for clinical translation (Bisht et al., 2026).

Despite these advances, several challenges remain. Standardization of culture protocols, incorporation of vascular and immune components, scalability, and genetic stability during long-term culture are critical areas requiring further development (He et al., 2025). Integration with artificial intelligence and multi-omics analyses promises to enhance organoid characterization, drug response prediction, and personalized therapy design (Balkhair and Albalushi, 2025; Yao et al., 2024). The convergence of organoid technology with bioengineering, genome editing, and computational modeling is poised to transform biomedical research and precision medicine, ultimately enabling patient-specific disease modeling and tailored therapeutic interventions (Tao et al., 2025; Luce and Duclos-Vallee, 2025).

In summary, organoid technology represents a paradigm shift in individualized medicine. It offers physiologically relevant, patient-specific models that recapitulate human organ complexity and disease heterogeneity. Multidisciplinary approaches have propelled organoids from experimental systems toward clinical translation, drug discovery, and regenerative therapies. Continued advancements and collaborative efforts are essential to overcome existing limitations and harness the full potential of organoids in personalized diagnostics and treatment strategies.

## 2 Technological foundations of organoids

### 2.1 Cell sources and culture systems

**FIGURE 1:**
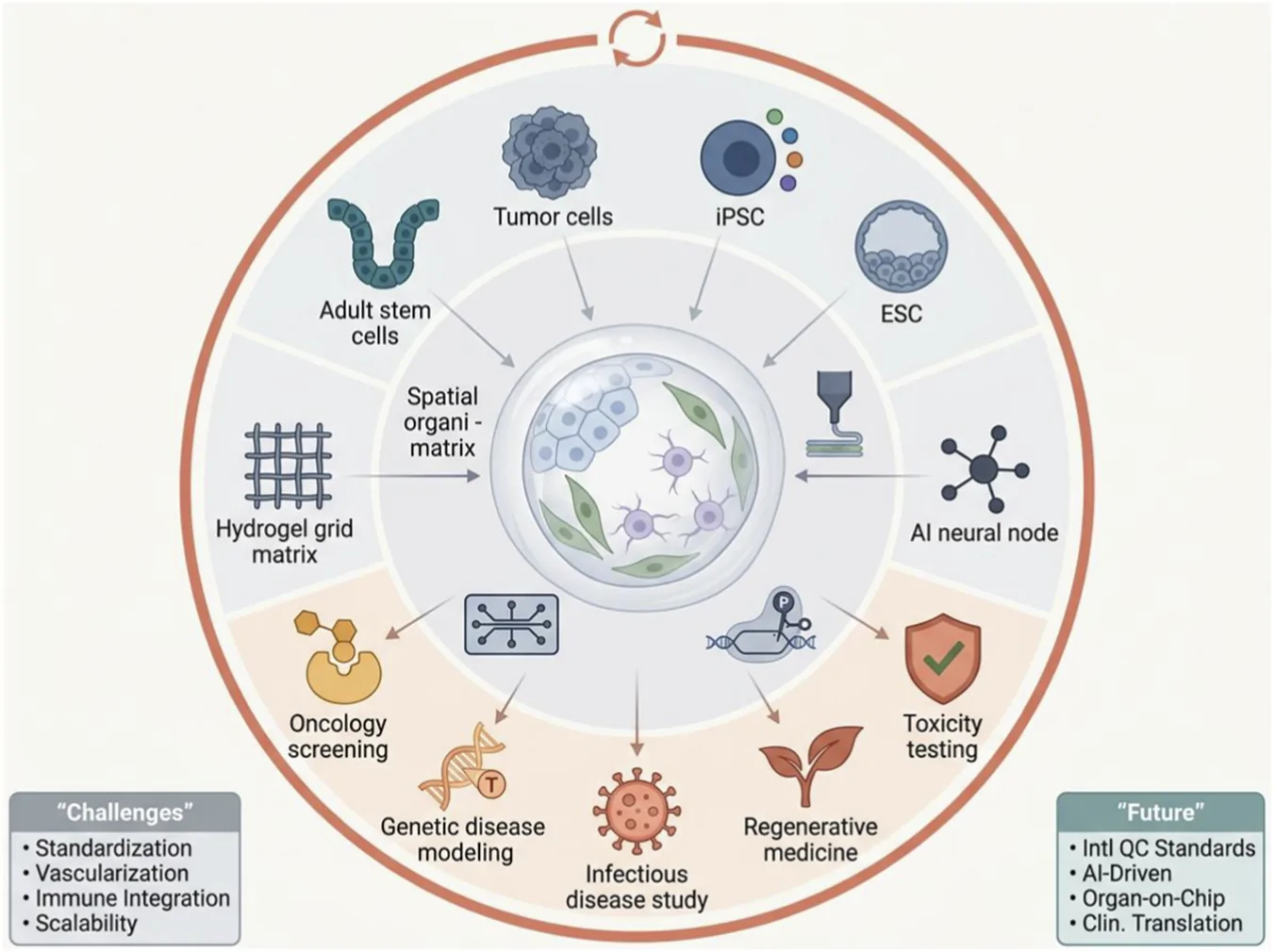
Technological foundations and application landscape of organoids in personalized medicine. Abbreviations; iPSCs, induced pluripotent stem cells; ESCs, embryonic stem cells; Note: Figures were created with the assistance of AI-based illustration tools and subsequently reviewed and edited by the authors for scientific accuracy.

The establishment of organoid culture systems has revolutionized biomedical research. These systems enable the *in vitro* recapitulation of three-dimensional architecture and key functions of native tissues. Organoids can be derived from various cellular sources (Table 1), including adult somatic stem cells, tumor cells, induced pluripotent stem cells (iPSCs), and embryonic stem cells (ESCs) (Figure 1). Each source offers unique advantages and challenges in culture conditions, differentiation potential, and application scope.

**TABLE 1:** Comparison of cell sources for organoid construction in personalized medicine applications.

Comparison dimension	ASC-derived organoids	iPSC-derived organoids	ESC-derived organoids
Cell source	Adult stem cells from patient tissue (intestine, liver, kidney, etc.)	Somatic cells reprogrammed to induced pluripotent stem cells	Embryonic stem cells derived from blastocyst inner cell mass
Establishment time	1–3 weeks from biopsy	4–8 weeks (reprogramming + differentiation)	4–6 weeks (differentiation only)
Throughput	Moderate to high (96-well compatible)	Low to moderate (labor-intensive differentiation)	Low to moderate (labor-intensive differentiation)
Cost per assay	Low to moderate	High (reprogramming, QC, karyotyping)	High (maintenance, ethical oversight)
Lineage fidelity	High (directly reflects source tissue)	Moderate (dependent on differentiation protocol)	Moderate (dependent on differentiation protocol)
Differentiation potential	Limited to source tissue lineage(s)	Broad (can generate multiple germ layers)	Broadest (pluripotent, can generate any cell type)
Developmental plasticity	Low (adult state, limited dedifferentiation)	High (can model organogenesis and development)	High (can model organogenesis and development)
Batch-to-batch variability	Moderate (patient-to-patient variation)	High (differentiation efficiency varies)	High (differentiation efficiency varies)
Tumor microenvironment	Absent (pure epithelial/stromal culture)	Can be engineered (organoid-on-chip, co-culture)	Can be engineered (organoid-on-chip, co-culture)
Immune components	Absent (pure epithelial/stromal culture)	Can incorporate iPSC-derived immune cells	Can incorporate ESC-derived immune cells
Maturation state	Adult-like, functionally mature	Fetal-like, often immature even after long culture	Fetal-like, often immature even after long culture
Genetic stability	Generally stable; accumulates somatic mutations with long-term culture	Risk of copy-number variations with prolonged culture	Generally stable with proper maintenance
Ethical considerations	Minimal (adult tissue biopsy)	Minimal (patient consent for reprogramming)	Significant (embryo destruction controversy)
Key advantage	Rapid, clinically relevant, patient-specific	Models development, disease onset, and multi-lineage processes	Highest differentiation potential, gold standard for pluripotency
Key limitation	Limited plasticity, cannot model developmental defects	Residual immaturity, long turnaround, high cost	Ethical constraints, same immaturity issues as iPSCs
Best application	Drug screening, precision medicine (cancer, genetic diseases)	Developmental disease modeling, drug discovery (multi-lineage effects)	Early development research, multi-lineage differentiation studies

Adult somatic stem cells from tissues such as nasal mucosa, lung, and bile duct have been widely used to generate organoids that preserve the histological and functional characteristics of the source tissue. Staged expansion-differentiation protocols enable the production of organoids that closely mimic native structures (Chiu et al., 2023; Wang K. et al., 2022; Li et al., 2022; Roos et al., 2020; Roos et al., 2021). The success of adult stem cell-derived organoids relies heavily on isolation techniques and maintenance of the stem cell niche, often involving extracellular matrix components and growth factors that simulate *in vivo* microenvironments.

Tumor tissue-derived organoids recapitulate the genetic, morphological, and pharmacological features of primary tumors. Patient-derived tumor organoids (PDTOs) can be established from diverse cancer types with high success rates and fidelity to parental tumors (Roos et al., 2021; Sui et al., 2025), enabling personalized drug screening and mechanistic studies. However, challenges such as tumor heterogeneity and microenvironmental factors remain. Co-culture systems incorporating cancer-associated fibroblasts or mesenchymal stromal cells have been developed to better mimic the tumor microenvironment (Sui et al., 2025; Naruse et al., 2021). Hypoxic culture conditions can select for specific subclones with distinct malignant traits (Kumano et al., 2024).

Induced pluripotent stem cells and embryonic stem cells serve as versatile sources for organoid generation due to their indefinite self-renewal and multilineage differentiation capacity. Protocols for differentiating iPSCs into nervous system organoids (Bhargava et al., 2022) and liver organoids (Nguyen et al., 2021) have been established, providing platforms for developmental biology and disease modeling.

Culture systems universally rely on extracellular matrix scaffolds and optimized media. Matrigel is the most common scaffold, but its undefined composition, batch variability, and animal origin pose challenges for reproducibility and clinical translation. Consequently, synthetic or biomimetic hydrogels with defined properties are being developed to support specific organoid types and functions (Luo et al., 2023; Zhao et al., 2025). Culture media typically contain growth factors and signaling modulators (e.g., Wnt agonists, R-spondin, Noggin, EGF, TGF-β inhibitors) to sustain stemness and regulate lineage commitment (Pleguezuelos-Manzano et al., 2020). Microenvironmental factors such as oxygen tension influence organoid characteristics (Kumano et al., 2024). Advanced culture systems employ dynamic devices like microfluidics and bioreactors to better emulate physiological conditions (Unagolla and Jayasuriya, 2022; Liu et al., 2024).

Overall, organoid sources include adult tissue-derived stem cells, tumor cells, and pluripotent stem cells, each requiring specific isolation and culture strategies. Culture systems integrate extracellular matrices, optimized media, and controlled environments to support organoid growth, differentiation, and functional maturation. Future improvements in synthetic matrices, culture standardization, and microenvironmental complexity will further enhance organoid fidelity and translational potential.

ASC-derived organoids offer high lineage fidelity and rapid establishment from patient tissues, but are restricted to source cell types and lack developmental plasticity. iPSC-derived organoids provide broader differentiation and developmental modeling, yet exhibit higher batch variability, residual immaturity, and prolonged differentiation protocols. ESC-derived organoids share similar advantages and limitations as iPSC counterparts, with additional ethical considerations from embryonic derivation.

Table 2 PDOs and patient derived xenograft (PDX) models. PDOs offer shorter establishment (1–3 weeks vs. 2–6 months), lower cost, compatibility with high throughput drug screening, and preservation of tumor genetic heterogeneity. However, they lack native tumor microenvironment, vasculature, and immune components. PDXs partially retain these features. PDXs better recapitulate drug pharmacokinetics and stromal interactions, but they also have limitations: species-specific stromal replacement over passages, higher cost, and lower throughput. Neither model alone fully recapitulates human tumor complexity. Complementary use may provide the most comprehensive preclinical assessment.

**TABLE 2:** Comparison of PDOs PDX models for preclinical drug testing.

Comparison dimension	Patient-derived organoids (PDOs)	Patient-derived xenograft (PDX) models
Establishment time	1–3 weeks from biopsy	2–6 months (sometimes longer for certain tumor types)
Success rate	50%–80% (tumor type dependent)	10%–60% (tumor type and passage number dependent)
Cost per model	Low to moderate	High (mouse husbandry, imaging, necropsy)
Throughput (drugs tested)	High (96-well to 384-well format feasible)	Low (one mouse = one data point; limited by mouse number)
Turnaround for drug testing	1–2 weeks after organoid establishment	4–8 weeks (tumor growth in mice before treatment)
Tumor genetic heterogeneity	Preserved (clonal diversity maintained in 3D culture)	Preserved initially, but selective pressure from mouse stroma causes drift over passages
Tumor microenvironment	Absent (pure tumor cell culture; can be engineered via co-culture)	Partially retained (mouse stromal, endothelial, and partial immune components; human immune cells lost over passages)
Immune system interaction	Absent (can be modeled with PBMC/organoid co-culture)	Mouse immune system (human immune cells replaced by mouse over passages); humanized PDX available but costly
Drug pharmacokinetics	Lacking (static culture; can be partially addressed with microfluidic systems)	Physiologically relevant (*in vivo* drug distribution, metabolism, clearance)
Stromal interaction	Absent (pure epithelial culture)	Mouse stromal cells replace human stroma over passages (species-specific stromal replacement)
Validation against patient outcome	Multiple prospective studies show ∼80–90% concordance (e.g., Veninga and Voest, 2021)	Established correlation, but prospective validation still limited
Inter-laboratory reproducibility	Moderate (protocol-dependent; Matrigel batch effects)	Moderate to high (mouse model standardization improving)
Scalability for large studies	High (cryopreservation, biobanking well established)	Low (mouse capacity constraint; expensive to scale)
Regulatory acceptance	No FDA-approved assay yet; multiple protocols in clinical trials	Established as pre-clinical model; regulatory pathway clearer than PDOs
Key strength	High-throughput, low cost, rapid, patient-tailored	*In vivo* relevance, PK/PD, stromal interaction
Key limitation	No microenvironment, no immune system, no PK/PD	Low throughput, high cost, long timeline, species differences
Complementary use	Ideal for first-line screening and hypothesis generation	Ideal for *in vivo* validation of organoid-screening hits and PK/PD studies

### 2.2 Phenotypic and functional characteristics

Organoids closely recapitulate the tissue architecture and cellular heterogeneity of their source organs. For instance, intestinal organoids derived from murine crypts faithfully mimic the *in vivo* intestinal epithelium (Kashfi et al., 2020), and cerebral organoids generated from human iPSCs demonstrate layered cytoarchitecture with distinct neuronal and glial populations (Ma et al., 2022). This cellular heterogeneity, supported by 3D cell-cell and cell-matrix interactions, is essential for modeling physiological and pathological processes.

In addition to structural fidelity, organoids exhibit gene expression profiles that closely mirror those of their tissues of origin. Transcriptomic analyses of patient-derived pancreatic ductal adenocarcinoma organoids show that hydrogel composition modulates organoid phenotype and gene expression (Sorzabal-Bellido et al., 2025). Gastric cancer organoids retain the histopathological and molecular features of primary tumors (Seidlitz et al., 2021), and normal tissue-derived organoids maintain stemness and differentiation markers (Ramezanpour et al., 2022). These findings collectively emphasize that organoids maintain a high degree of molecular fidelity.

Functionally, organoids simulate key physiological processes including secretion, metabolism, and drug responses. Liver organoids derived from iPSCs demonstrate albumin and urea production, fatty acid metabolism, and disease-specific phenotypes (Kiyuna et al., 2025). Intestinal organoid-derived epithelial cells exhibit nutrient uptake and cytochrome P450 induction (Takahashi et al., 2022). Neural organoids cultured with vascular-inspired scaffolds show enhanced neuronal maturation and network activity (Cai et al., 2025). These functional attributes enable organoids to serve as predictive models for drug screening and toxicity testing. Functional heterogeneity highlights the importance of phenotypic characterization for precision medicine (Huang et al., 2024).

Organoids’ 3D architecture, heterogeneity, gene expression, and physiological functions make them indispensable in biomedical research. Standardizing culture systems and improving scalability will be critical to fully harness their clinical potential.

### 2.3 Technological advances driving organoid applications

The integration of advanced technologies has significantly propelled organoid utility in personalized medicine (Figure 1). High-throughput screening (HTS) techniques combined with organoid drug sensitivity assays enable rapid, efficient screening of large compound libraries to assess drug efficacy and cytotoxicity in a patient-specific context. This approach enhances predictive accuracy of drug responses and facilitates precision medicine strategies (Ren et al., 2022). Microfluidic technologies and automation have further increased throughput and reproducibility, addressing limitations related to culture variability and scalability (Liu et al., 2024).

Gene editing technologies, particularly CRISPR/Cas9, allow precise introduction of mutations, correction of genetic defects, or insertion of reporter genes within a physiologically relevant 3D context. This capability is instrumental for studying molecular mechanisms underlying development, tumorigenesis, and genetic disorders, and for engineering organoids for regenerative medicine (Chen et al., 2021; Wu et al., 2023). CRISPR/Cas9 enables the generation of isogenic control lines, which are critical for delineating genotype-phenotype relationships.

Single-cell sequencing and multi-omics technologies have unveiled the complex cellular heterogeneity within organoids. Single-cell RNA sequencing (scRNA-seq) reveals diverse cell subtypes and maturation states (Gu et al., 2023). Integrated multi-omics data provide insights into regulatory networks and cell-cell interactions. These high-resolution analyses support the identification of rare cell populations and therapeutic targets, advancing studies of tumor microenvironment heterogeneity and immunotherapy (Varinelli et al., 2025).

Together, the convergence of HTS, gene editing, and single-cell multi-omics technologies has expanded organoid capabilities for accurate disease modeling, personalized drug testing, and mechanistic studies at cellular resolution. Continued integration and refinement promise to overcome current limitations in heterogeneity and scalability, accelerating clinical translation.

### 2.4 Standardization and quality control

Standardization of cell sources and culture protocols is foundational to organoid reproducibility and reliability. Organoids derived from various origins (PSCs, adult stem cells, progenitor cells, or differentiated cells) inherently introduce variability. Standardized protocols for cell isolation, expansion, and differentiation have been developed to improve consistency (Devasahayam Arokia Balaya et al., 2025). Bioprinting technologies enable precise spatial arrangement of multiple cell types, reducing heterogeneity and promoting consistent self-assembly (Tong et al., 2025).

Quality control (QC) metrics encompass morphological, functional, and genetic stability assessments. Morphological evaluation includes size, structural integrity, and cellular composition; for brain organoids, Feret diameter correlates with quality (Boerstle et al., 2025). Functional assays assess organ-specific activities, and genetic stability is monitored over passages. Standardized quality attributes facilitate comparability and regulatory acceptance, particularly for tumor organoid biobanks (Fan et al., 2026; Ahn et al., 2024). Non-invasive, automated monitoring using high-content imaging and machine learning enables real-time quality assessment without compromising viability (Matsumoto et al., 2025).

Inter-laboratory variability remains a challenge. Comprehensive guidelines for organoid manufacturing and application have been developed (Lim et al., 2024; Lee H. A. et al., 2024; Lee H. et al., 2024; Kwak et al., 2024; Lee S. et al., 2024), and biobanks with standardized, quality-controlled lines contribute to resource availability (Xie et al., 2023). Microfluidic and bioreactor systems reduce variability and promote maturation (Charles et al., 2025). AI-based image analysis pipelines enable non-invasive monitoring of morphology and growth dynamics, and machine learning models predict organoid formation efficiency (Matsumoto et al., 2025; Deininger et al., 2023). Bioprinting further reduces batch-to-batch variability and enables scalable production (Tong et al., 2025; Kang et al., 2024).

Standardizing cell sources, culture protocols, and QC (morphological, functional, and genetic) are essential for reproducible organoid generation. Advanced bioengineering, microfluidic culture, and AI-driven quality assessments enhance consistency and reliability, accelerating clinical translation.

## 3 Organoids in oncology: from model to clinic

### 3.1 Establishment and fidelity of tumor organoids

The establishment of tumor organoids derived from patient tumor tissues has become a transformative approach for cancer research. These organoids recapitulate the three-dimensional architecture, genetic heterogeneity, and phenotypic characteristics of the original tumors. The success rate of generating tumor organoids varies depending on tumor type, tissue quality, and culture conditions, but recent advances have improved efficiency across multiple cancer types. For example, pancreatic cancer organoids have been established with success rates exceeding 70%, preserving the genetic landscape and enabling functional drug testing relevant to patient-specific responses (Hu et al., 2025). Similarly, colorectal cancer (CRC) organoids maintain intratumoral heterogeneity and molecular subtypes. Orthotopic transplantation models preserve histological and transcriptomic features of the parental tumors (Jesinghaus et al., 2026). The ability to culture organoids from both treatment-naïve and treated tumors has provided insights into therapy resistance mechanisms. For instance, pancreatic ductal adenocarcinoma organoids derived from patients treated with neoadjuvant FOLFIRINOX recapitulated resistance profiles observed clinically (Farshadi et al., 2021).

The phenotypic stability of tumor organoids during long-term culture is critical for their utility in translational research. Studies have shown that organoids retain key driver mutations and morphological features over multiple passages. For example, small cell lung cancer organoids maintained genetic profiles and phenotypic heterogeneity during extended expansion, reflecting differential chemotherapeutic responses observed in patients (Choi et al., 2021). Moreover, co-culture systems have enhanced the preservation of tumor microenvironment (TME) components, such as fibroblasts, within organoid cultures, better mimicking *in vivo* conditions (Sui et al., 2025). These advanced culture systems have facilitated the study of tumor-stroma interactions and drug resistance mechanisms, underscoring the importance of microenvironmental factors in tumor biology.

**FIGURE 2:**
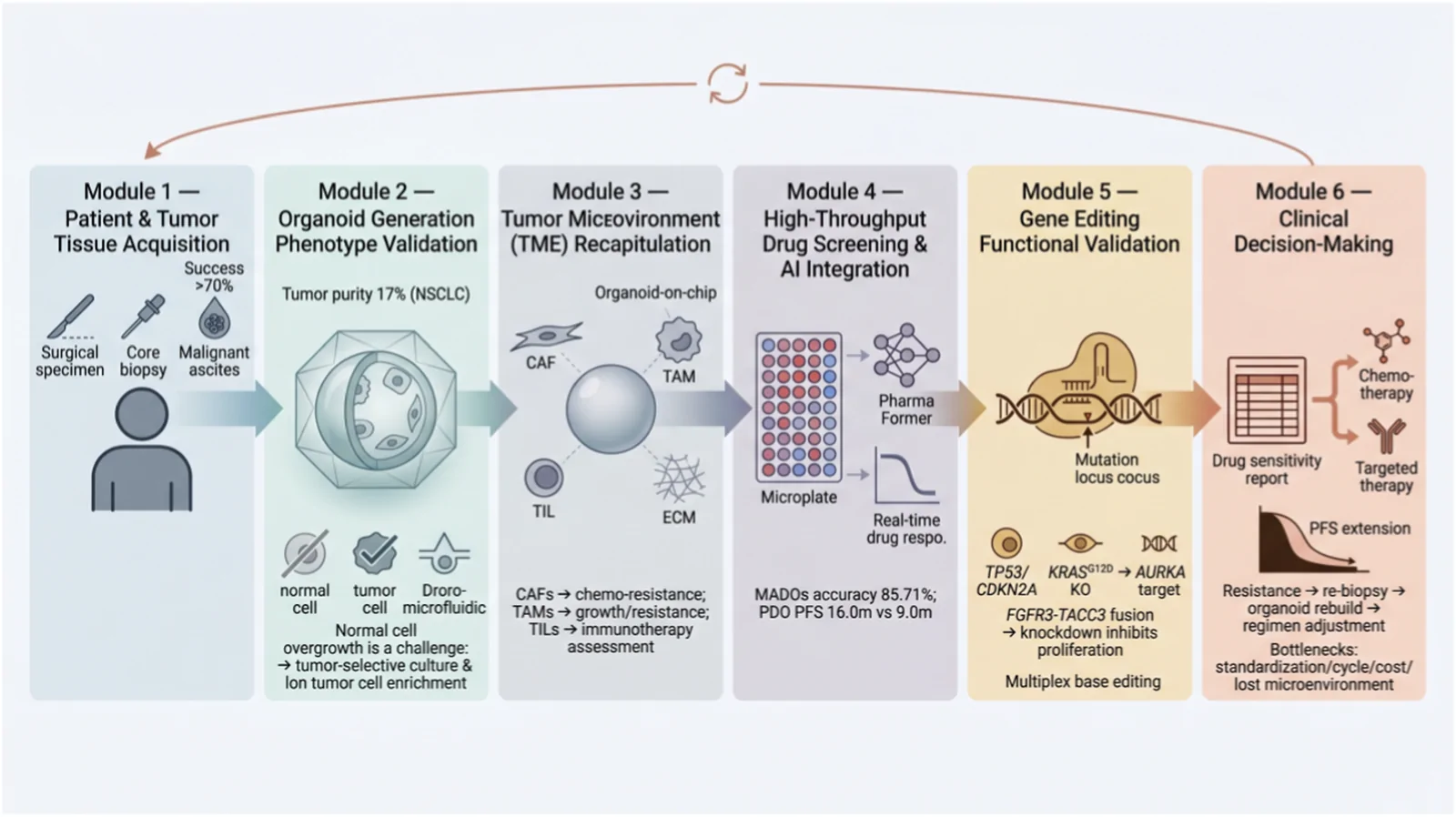
From biopsy to bedside: the tumor organoid precision medicine workflow. Note: This figure was created with the assistance of AI-based illustration tools and subsequently reviewed and edited by the authors for scientific accuracy.

An important aspect of tumor organoid establishment is preventing overgrowth by normal cells, which can compromise tumor purity. In non-small cell lung cancer (NSCLC), only about 17% of organoids derived from intrapulmonary lesions were confirmed to be pure tumor organoids due to overgrowth of normal airway organoids (Dijkstra et al., 2020). Strategies such as tumor-selective media and exclusion of enzymatic dissociation steps have been employed to enrich tumor cells and maintain original tumor histology and genetic alterations, as demonstrated in meningioma organoids (Kim et al., 2024). Additionally, PDX-derived organoids provide renewable sources of tumor cells that maintain platinum sensitivity profiles consistent with patient responses in ovarian cancer, offering a scalable model for drug screening (Figure 2).

The integration of cutting-edge technologies, including microfluidic droplet encapsulation and 3D bioprinting, has further enhanced the reproducibility and phenotypic fidelity of tumor organoids. Droplet-engineered organoids exhibit improved tumor purity and modeling success, facilitating drug screening applications (Zhang et al., 2024). Biomimetic 3D-bioprinted organoids replicate native tumor biophysical and molecular characteristics more faithfully than traditional Matrigel-cultured organoids. These innovations support the generation of organoids that closely mimic patient tumors, providing robust platforms for personalized medicine.

In summary, the establishment of tumor organoids with high success rates and phenotypic stability has been achieved across various cancer types. These organoids preserve the genetic and histological features of their parental tumors and maintain tumor heterogeneity, which is crucial for modeling disease progression and therapeutic responses. The incorporation of tumor microenvironment components and the application of advanced culture technologies further enhance the physiological relevance of organoid models, positioning them as indispensable tools for cancer research and precision oncology.

### 3.2 Recapitulating tumor microenvironment and drug resistance

Tumor organoids have emerged as advanced three-dimensional culture models that preserve tumor cell heterogeneity. They also enable recapitulation of complex tumor microenvironments *in vitro*. The TME comprises cancer-associated fibroblasts (CAFs), immune cells, extracellular matrix (ECM), and vasculature. It plays a pivotal role in tumor progression, metastasis, and therapeutic resistance. Traditional two-dimensional cultures and even some organoid models lack the full complement of these components, limiting their ability to accurately mimic *in vivo* tumor biology (Guo et al., 2024).

Recent advances have focused on integrating multiple cellular components into organoid cultures to better simulate the TME. Co-culture systems combining tumor organoids with CAFs have demonstrated enhanced tumor growth and conferred resistance to chemotherapeutic agents such as sorafenib and 5-fluorouracil, highlighting the trophic and protective roles of stromal cells (Liu et al., 2021). Similarly, inclusion of tumor-associated macrophages (TAMs) in co-culture with cholangiocarcinoma organoids promoted organoid growth and chemotherapy resistance, underscoring the immunomodulatory influence of TAMs within the TME (Guo et al., 2024). These models enable dissection of paracrine signaling pathways and cell-cell interactions that drive tumor progression and drug resistance.

Immune components are increasingly incorporated into organoid systems to study tumor-immune interactions and immunotherapy responses. For example, co-culture of patient-derived colorectal cancer organoids with autologous tumor-infiltrating lymphocytes (TILs) allows assessment of TIL-mediated cytotoxicity and prediction of immunotherapy efficacy (Feng et al., 2025). Melanoma patient-derived organoids have been employed to evaluate the cytotoxicity of γδ T-cells, facilitating exploration of cellular therapies in a physiologically relevant context (Goncalves et al., 2024). Additionally, organoid models have been used to investigate metabolic reprogramming in immune cells that enhances checkpoint blockade efficacy, demonstrating their utility in immunometabolism studies.

The physical and biochemical properties of the ECM are critical determinants of tumor behavior. Organoid cultures embedded in biomimetic matrices such as Matrigel or alginate hydrogels provide structural support that influences organoid morphology, mechanical forces, and drug responses (Fang et al., 2021). Microfluidic organoid-on-chip platforms enable incorporation of vascular networks and real-time monitoring of secreted biomarkers, enhancing physiological relevance and translational potential (Ruan et al., 2026). These platforms facilitate the study of tumor angiogenesis, nutrient gradients, and drug delivery dynamics.

Tumor heterogeneity, both inter- and intra-tumoral, poses significant challenges for effective cancer treatment. Patient-derived organoids capture this heterogeneity, as evidenced by studies in colorectal, breast, and prostate cancers where organoids retained diverse cellular states and molecular profiles reflective of the original tumors (Ludwik et al., 2024; Li et al., 2024; Song et al., 2022). Single-cell RNA sequencing of organoids has revealed distinct subpopulations, enabling the study of tumor heterogeneity (Song et al., 2022; Liu et al., 2020). This cellular diversity supports the use of organoids for personalized drug screening and biomarker discovery.

Despite these advances, challenges remain in fully recapitulating the complexity of the TME, such as modeling vascularization, immune cell diversity, and systemic interactions. Emerging methods combining organoid technology with gene editing, synthetic biology, and microengineering hold promise for overcoming these limitations and creating dynamic, living biosensors of tumor biology (Zhou et al., 2026; Yang et al., 2023). Continued development of standardized protocols and integration with high-throughput screening technologies will accelerate the application of organoid models in precision oncology.

Overall, tumor organoids represent a versatile and physiologically relevant platform for modeling the tumor microenvironment and heterogeneity *in vitro*. Incorporation of stromal and immune components, advanced biomaterials, and microfluidic systems enhances their utility for studying tumor biology, drug resistance, and immunotherapy. These sophisticated models provide critical insights into tumor-stroma-immune interactions and facilitate the development of personalized therapeutic strategies.

### 3.3 High-throughput drug sensitivity testing and AI integration

Patient-derived organoids faithfully recapitulate the histological, genetic, and phenotypic features of original tumors, making them highly suitable for drug sensitivity and resistance prediction. In colorectal cancer, PDOs established from heavily pre-treated patients preserve genomic and phenotypic characteristics, This enables drug panel testing that reveals differential responses to agents like oxaliplatin and palbociclib. Proteotranscriptomic analyses have elucidated distinct molecular pathways associated with drug resistance. These include enrichment of t-RNA aminoacylation and a metabolic shift toward oxidative phosphorylation in oxaliplatin non-responders. These findings highlight potential biomarkers for predicting therapeutic outcomes (Wang et al., 2023).

In gastrointestinal cancers with peritoneal metastasis, malignant ascites-derived organoids (MADOs) demonstrate high culturing success rates and morphological consistency with *in vivo* tumors. Drug sensitivity assays on MADOs show strong correlation with clinical treatment efficacy, with accuracy rates up to 85.71%, indicating their potential as predictive tools for chemotherapy response (Yu et al., 2025). In stage IV colorectal cancer, PDO-based drug tests predict progression-free survival (PFS) after surgery. Patients classified as drug-sensitive exhibited a median PFS of 16.0 months versus 9.0 months in the drug-resistant group. PDO drug resistance was identified as an independent predictor of poor prognosis (Wang et al., 2023).

Beyond gastrointestinal tumors, artificial intelligence has enhanced the predictive power of organoid-based drug response models. PharmaFormer, a transformer-based AI model trained on 2D cell line data and fine-tuned with organoid pharmacogenomic profiles, improves the accuracy of clinical drug response predictions across cancer types (Zhou Y. et al., 2025). Integration of AI with organoid platforms enables rapid, robust prediction of patient-specific drug efficacy.

However, the integration of AI into organoid-based drug screening faces several critical limitations. First, AI predictions lack interpretability: deep learning models often operate as “black boxes,” hindering clinicians’ understanding of the biological rationale behind drug responses. This undermines trust and delays regulatory approval. Second, AI models demand large, well-annotated training datasets, but organoid drug response data remain scarce, heterogeneous across laboratories, and lacking standardized quality metrics, risking overfitting and poor generalizability. Third, integrating multi-omics data (genomic, transcriptomic, proteomic, morphological) with organoid screens poses challenges in data normalization, missing data handling, and reconciling discordant signals across layers. Fourth, regulatory frameworks are still evolving; the FDA’s SaMD pathway and the EU AI Act impose stringent requirements for validation, transparency, and post-market surveillance that current organoid-AI pipelines have yet to fully address.

Optimization of organoid culture systems has advanced drug screening applications. A dynamic culture system for breast cancer organoids provides continuous nutrient supply, shortening culture periods without compromising histological features or drug sensitivity profiles (Yang et al., 2025). High-throughput screening platforms combined with kinetic monitoring software, such as OrBITS, enable real-time, label-free assessment of organoid growth and drug response, improving consistency and scalability (Deben et al., 2023).

Organoid models have also been instrumental in optimizing multi-drug combination therapies. Screening large drug libraries on ovarian cancer organoids identified agents like afatinib that exhibit additive effects with carboplatin, suggesting novel combination regimens to overcome resistance (Ito et al., 2023). In pancreatic ductal adenocarcinoma, single-organoid analyses reveal intra-patient heterogeneity in drug response and invasive phenotypes, enabling identification of resistant subclones (Le Compte et al., 2023).

Organoid models facilitate the study of drug penetration and metabolism. Renal cell carcinoma organoids have been used to evaluate pharmacokinetics, including drug penetration and off-target toxicity, within a spatially organized tumor microenvironment (Gao et al., 2025). In hepatobiliary and pancreatic cancers, patient-derived organoids surpass traditional models in predicting drug response, although challenges remain in simulating tumor angiogenesis and the microenvironment (Hu et al., 2025).

Overall, patient-derived organoids represent a versatile platform for predicting drug sensitivity and resistance. Their ability to preserve tumor heterogeneity, coupled with advances in multi-omics integration, AI-assisted analysis, dynamic culture systems, and high-throughput screening, enhances predictive accuracy and applicability in personalized medicine.

### 3.4 Gene editing-assisted tumor mechanism research

The integration of gene editing technologies, particularly CRISPR-Cas systems, with organoid models has revolutionized tumor biology. It enables precise functional validation of tumor driver genes. Organoids derived from patient tumors preserve genetic heterogeneity and microenvironmental context, providing an ideal platform for dissecting gene function in a physiologically relevant setting. For instance, CRISPR-Cas9-mediated knockout of TP53 and CDKN2A in gastroesophageal junction organoids induced dysplastic and neoplastic features, recapitulating early tumorigenic events and revealing metabolic and epigenomic alterations associated with tumor progression (Zhao et al., 2022). In breast cancer research, *in vivo* genome-wide CRISPR screens coupled with *ex vivo* editing of mammary epithelial organoids identified tumor suppressor genes including Axin1 and Prkar1a that cooperate with Trp53 loss to accelerate tumorigenesis (Heitink et al., 2022).

Beyond functional validation, gene editing has facilitated the molecular dissection of drug resistance mechanisms. Colorectal cancer organoids subjected to chemotherapy and EGFR inhibition developed drug tolerance through transcriptomic adaptations. Acquisition of KRASG12D mutations further enhanced resistance. CRISPR editing revealed that inhibition of Aurora kinase A (AURKA) could restore apoptosis in drug-tolerant organoids, suggesting AURKA as a therapeutic target to overcome chemoresistance (Boos et al., 2022). CRISPR-Cas13a-mediated knockdown of the FGFR3-TACC3 fusion gene in bladder cancer organoids significantly inhibited proliferation, indicating that gene editing can target fusion oncogenes contributing to resistance (Wang Y. et al., 2024).

Gene editing also accelerates the discovery of novel therapeutic targets. Multiplex base editing in adult stem cell-derived organoids enabled simultaneous introduction of multiple cancer-associated mutations, modeling colorectal tumorigenesis in a single step and revealing potential vulnerabilities (Geurts et al., 2023). CRISPR knock-in strategies have been developed to fluorescently label and isolate specific tumor cell subpopulations within organoids, allowing lineage tracing and identification of targets linked to tumor heterogeneity (Cortina and Canellas-Socias, 2024). In immunotherapy, CRISPR-based editing of immune checkpoint genes in tumor-infiltrating lymphocytes improved their expansion, cytokine production, and tumor-killing ability (Hafezi et al., 2025).

The convergence of gene editing and organoid technologies also enables exploration of tumor-microenvironment interactions. Tumor organoids co-cultured with immune and stromal cells, combined with CRISPR-mediated modifications, can recapitulate complex interplay within the TME, offering insights into immune evasion and resistance (Si et al., 2025). Gene-editing nanoplatforms enhance delivery efficiency, which is critical for clinical translation (Ji et al., 2022; Liu C. et al., 2025).

Gene editing-assisted organoid research has advanced tumor biology by enabling functional validation of cancer drivers, elucidation of resistance mechanisms, and identification of therapeutic targets in physiologically relevant context. Further refinement of editing tools and delivery methods will enhance these platforms.

### 3.5 Clinical translation: successes, barriers, and strategies

The clinical translation of tumor organoids has demonstrated promising successes in guiding personalized treatment. PDTOs faithfully recapitulate histopathological, genetic, and phenotypic characteristics of original tumors. This enables individualized drug sensitivity testing that correlates with patient responses. Studies in colorectal cancer have shown that PDTOs can predict responses to irinotecan-based neoadjuvant chemoradiotherapy (Meier et al., 2022; Sanchez et al., 2024). Lung cancer organoids simulate tumor heterogeneity and metastatic behavior, providing platforms for drug screening and immunotherapy evaluation (Zeng et al., 2023; Ma J. et al., 2025). Rare cancer subtypes, such as hepatocellular carcinoma with neuroendocrine differentiation, have benefited from rapid organoid establishment enabling *in vitro* drug testing that informed personalized therapeutic decisions (Meier et al., 2022).

Despite these advances, several challenges impede widespread clinical application. Ethical considerations arise regarding patient consent and data privacy, particularly as biobanking platforms evolve to include blockchain-based patient engagement systems (Sanchez et al., 2024). Time constraints are significant; organoid establishment often requires weeks, which may not align with urgent clinical timelines. Single-cell derived organoids have been developed to accelerate generation and reduce turnaround time (Gao et al., 2021). Technical limitations include variability in culture success rates, lack of standardized protocols, and insufficient recapitulation of the TME, particularly immune and stromal components (Foo et al., 2022; Zhang et al., 2026). High costs and labor-intensive nature hinder scalability.

Robust data management and multi-center collaboration are needed. Large-scale biobanks of patient-derived organoids facilitate drug screening and mechanistic studies (Kumari et al., 2022). Combining organoid platforms with AI and multi-omics analyses offers promising avenues to streamline data interpretation, optimize drug screening, and predict therapeutic responses with higher accuracy (Bai et al., 2024; Ma W. et al., 2025).

Several practical barriers limit the clinical application of organoid guided decisions. Turnaround time is a major bottleneck: establishment takes 2–6 weeks, plus 1–2 weeks for drug sensitivity testing, often too slow for urgent decisions in aggressive cancers. Reproducibility suffers from variability in tissue sourcing, enzymatic dissociation, and Matrigel batch composition, causing inconsistent drug response predictions. Furthermore, the lack of standardized quality metrics (e.g., viability thresholds, consensus response criteria, reference controls) impedes inter-laboratory comparability and regulatory acceptance. Until these issues are addressed in prospective multicenter trials using standardized protocols, organoid based drug testing should remain complementary, not a standalone clinical tool.

Despite these challenges, organoid technology has shown remarkable potential in personalized medicine. However, significant challenges related to ethics, time, cost, technical limitations, and data integration remain. Addressing these issues through technological innovation, standardization, and collaborative frameworks will be pivotal in realizing the full clinical utility of organoids for individualized patient care.

## 4 Research progress of organoids in hereditary and infectious diseases

### 4.1 Construction of genetic disease models

**FIGURE 3:**
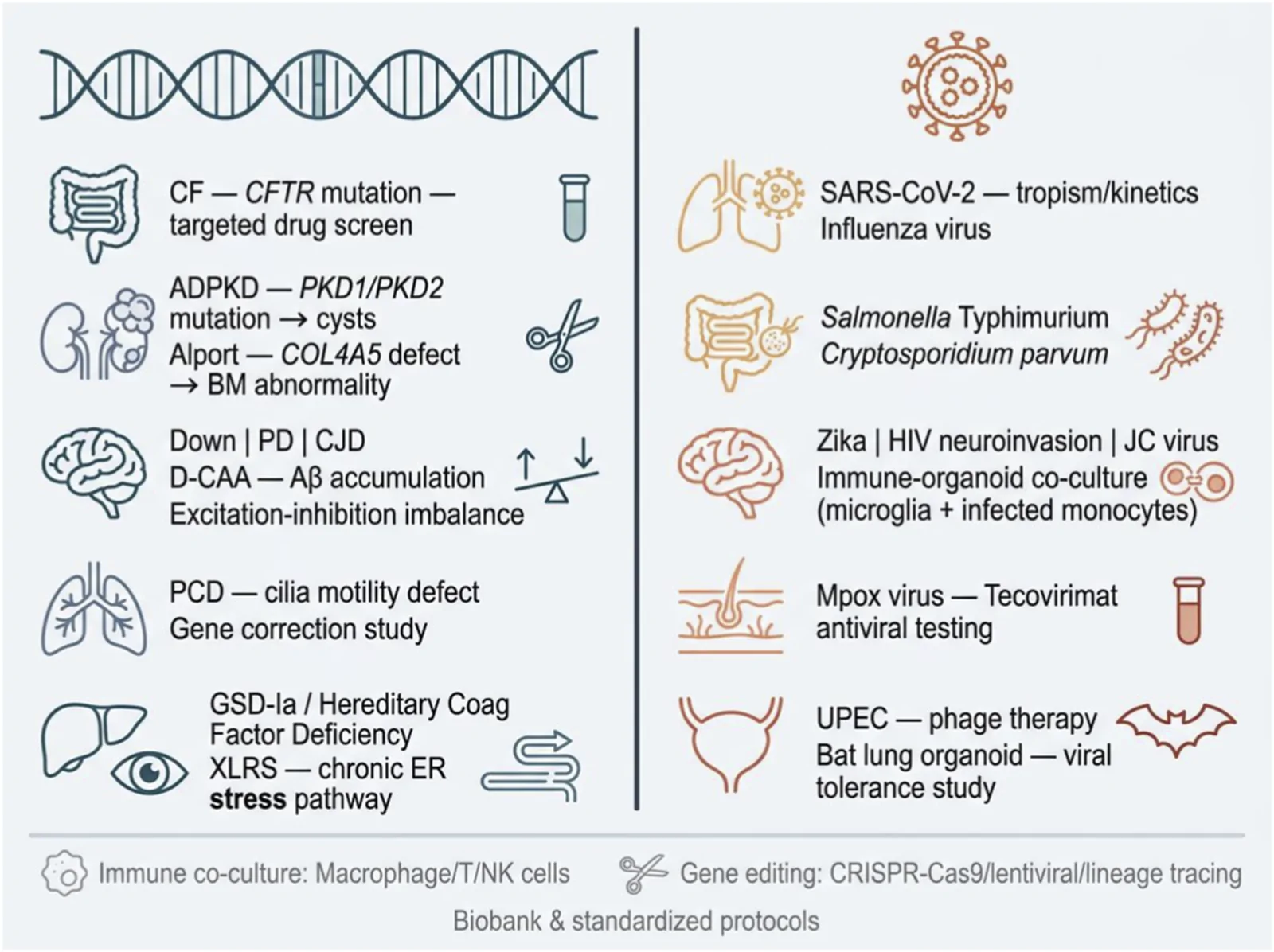
Genetic diseases and infectious diseases: the dual-track application of organoid models Abbreviations: CF, cystic fibrosis; CFTR, cystic fibrosis transmembrane conductance regulator; ADPKD, autosomal dominant polycystic kidney disease; UPEC: Uropathogenic Escherichia coli; CRISPR, Clustered Regularly Interspaced Short Palindromic Repeats; SARS-CoV-2, severe acute respiratory syndrome coronavirus 2; HIV, human immunodeficiency virus; NK, natural killer. Note: This figure was created with the assistance of AI-based illustration tools and subsequently reviewed and edited by the authors for scientific accuracy.

The advent of organoid technology has revolutionized the modeling of genetic diseases. It provides three-dimensional *in vitro* systems that faithfully recapitulate key structural, functional, and genetic features of human organs (Figure 3). Among genetic disorders, cystic fibrosis (CF) stands out as a prototypical disease for which organoid models have been extensively developed. CF is caused by mutations in the CFTR gene, leading to defective chloride ion transport and multisystemic manifestations, primarily affecting the lungs and digestive tract. Patient-derived organoids, especially intestinal organoids, have been generated from pluripotent stem cells or adult stem cells (ASCs), preserving patient-specific genetic mutations and phenotypes (Wang Q. et al., 2022). These organoids exhibit characteristic disease traits such as altered ion transport and mucus production, enabling the study of disease mechanisms and facilitating personalized drug screening. The ability to culture organoids from patients carrying different CFTR mutations allows assessment of mutation-specific therapeutic responses, thereby advancing precision medicine in CF (Mijnders et al., 2022).

Beyond CF, kidney organoids have emerged as powerful models for hereditary kidney diseases, including autosomal dominant polycystic kidney disease (ADPKD) and Alport syndrome. ADPKD, caused mainly by mutations in PKD1 and PKD2, is characterized by progressive cyst formation in renal tubules. Kidney organoids derived from disease-specific human iPSCs or genetically edited PSCs have successfully recapitulated cystogenesis *in vitro*. For instance, CRISPR-Cas9-mediated knockout of PKD1 in hiPSCs followed by differentiation into kidney organoids results in cyst-like structures, mimicking the pathophysiology of ADPKD (Shamshirgaran et al., 2021; Shimizu et al., 2020). Similarly, kidney organoids generated from Alport syndrome patient-derived iPSCs exhibit defective collagen IV α5 chain expression and basement membrane abnormalities, providing a platform for investigating disease mechanisms and testing therapeutic interventions (Hirayama et al., 2023; Yabuuchi et al., 2024). These models underscore the capacity of organoids to model monogenic kidney disorders with high fidelity.

The utility of organoids extends to neurological genetic diseases. Cerebral organoids derived from patient-specific iPSCs harboring mutations associated with disorders such as Down syndrome, Parkinson’s disease, and Creutzfeldt-Jakob disease have been established. These cerebral organoids reveal altered neuronal network activity, excitatory-inhibitory imbalances, and disease-specific pathophysiological features, despite lacking overt neurodegenerative pathology at early stages (Foliaki et al., 2021). Moreover, brain organoids modeling Dutch-type cerebral amyloid angiopathy (D-CAA), a hereditary form of cerebral amyloid deposition, demonstrate amyloid beta accumulation and dysregulated neuronal and astrocytic gene expression, opening avenues for mechanistic studies and therapeutic screening (Daoutsali et al., 2022). Incorporation of gene editing technologies such as CRISPR/Cas9 into brain organoid protocols further enables correction or introduction of disease-causing mutations, enhancing the precision of genetic disease modeling (Fischer et al., 2019).

In respiratory genetic diseases, airway organoids derived from patients with primary ciliary dyskinesia (PCD) have been successfully generated, preserving the motile cilia defects characteristic of the disease. These organoids allow detailed phenotypic analyses of ciliary beating abnormalities and facilitate gene correction studies, exemplifying the translational potential of organoid models in hereditary airway disorders (van der Vaart et al., 2021). Similarly, respiratory organoids have been used to model cystic fibrosis and primary ciliary dyskinesia (Deguchi and Takayama, 2023).

The generation of organoids from patient-derived or genetically engineered stem cells has also been applied to liver genetic diseases. Liver organoids maintain genetic and phenotypic hallmarks of hereditary liver disorders such as glycogen storage disease type Ia and inherited bleeding disorders involving coagulation factor deficiencies. These models provide platforms for studying disease pathogenesis (Mun et al., 2023; Roman et al., 2023). Optimized protocols have enhanced the scalability and reproducibility of liver organoid cultures, enabling their use in personalized medicine (Bai et al., 2024).

Gene editing technologies have been integrated with organoid culture systems to validate gene function and model genetic mutations *in vitro*. For example, inducible Cas9-expressing hiPSC lines have facilitated efficient CRISPR-mediated gene editing in kidney organoids, accelerating target validation and disease modeling without laborious clonal selection (Shamshirgaran et al., 2021). Lentiviral transduction methods optimized for organoid cultures further enhance genetic manipulation capabilities, broadening the scope of disease modeling and drug screening (Rathje et al., 2025).

Multi-omics approaches have further deepened our understanding of genetic disease mechanisms within organoid models. In X-linked retinoschisis models, multi-modal transcriptomic analyses of patient-derived retinal organoids uncovered chronic ER stress and eIF2 signaling as conserved pathological pathways. These findings were subsequently validated by proteomics and functional assays (Chien et al., 2025). Such integrative analyses highlight the power of multi-omics to uncover convergent disease mechanisms and identify therapeutic targets.

Overall, organoid models of genetic diseases provide unprecedented opportunities to dissect molecular mechanisms, validate gene functions, and screen therapeutic compounds in a patient-specific manner. Their ability to recapitulate complex tissue architecture and maintain genetic fidelity positions them as superior alternatives to traditional 2D cultures and animal models. However, challenges remain in achieving full maturation, vascularization, and immune system integration within organoids, which are essential for accurately modeling disease phenotypes and therapeutic responses. Addressing these limitations will further enhance the translational value of organoids in genetic disease research (Peng et al., 2022). Continued refinement of organoid technologies, combined with advances in single-cell sequencing and gene editing, promises to accelerate the development of personalized treatments and regenerative medicine applications (Li T. et al., 2025).

### 4.2 Infectious disease organoid models

Organoid models have emerged as transformative platforms for studying infectious diseases. They provide physiologically relevant three-dimensional tissue structures that closely recapitulate human organ architecture and function (Figure 3). Traditional two-dimensional cell cultures and animal models often fail to mimic the complex cellular heterogeneity and microenvironment of human tissues, limiting their translational relevance in understanding pathogen-host interactions and drug responses. Organoids derived from pluripotent stem cells or adult stem cells possess self-renewal and self-organization capacities, enabling the generation of miniaturized organ-like structures that faithfully reproduce key features of *in vivo* organs (Blutt and Estes, 2022; Shpichka et al., 2022). These models have been successfully established for various human tissues including lung, intestine, brain, liver, skin, and kidney. They have been applied to investigate diverse infectious agents such as viruses, bacteria, fungi, and protozoa.

The ability of organoids to simulate pathogen infection mechanisms has been demonstrated in multiple systems. Lung organoids have been utilized to model respiratory infections by SARS-CoV-2 and influenza viruses, revealing viral tropism, replication dynamics, and host immune responses that are difficult to capture in 2D cultures or animal models (Li J. et al., 2025; Peng et al., 2022). Intestinal organoids have provided insights into enteric infections caused by pathogens like *Salmonella Typhimurium* and Cryptosporidium parvum (Yan et al., 2024; Varegg et al., 2025). Brain organoids have enabled the study of neurotropic viruses such as Zika virus, HIV, and JC virus, allowing investigation of viral neuroinvasion, tropism, and neuropathogenesis in a human-relevant context (Su et al., 2021; Donadoni et al., 2024). These organoid infection models facilitate detailed analyses of pathogen life cycles, host cell susceptibility, and molecular pathogenesis, which are critical for developing targeted interventions.

Beyond modeling infection mechanisms, organoids serve as valuable platforms for high-throughput screening of anti-infective agents and evaluation of therapeutic efficacy. The 3D architecture and multicellular composition of organoids better reflect drug penetration, metabolism, and cytotoxicity compared to conventional models. For example, human skin organoids infected with mpox virus have been used to assess the antiviral drug tecovirimat, demonstrating its ability to inhibit viral replication and modulate host transcriptomic responses (Li et al., 2023). Similarly, bladder organoids infected with uropathogenic *Escherichia coli* have been employed to evaluate bacteriophage therapy, showing reduced bacterial burden and inflammation. These applications underscore the potential of organoids in accelerating preclinical drug development and personalized medicine approaches for infectious diseases.

A critical advancement is the integration of immune components into organoid cultures to better mimic the *in vivo* immune microenvironment during infection. Co-culture systems combining organoids with immune cells such as macrophages, T cells, or natural killer (NK) cells have been established to study immune-pathogen interactions and host defense mechanisms. Immune-competent brain organoids containing microglia and infected monocytes have been developed to investigate HIV neuroinvasion and latency within the central nervous system (Dos Reis et al., 2024). Moreover, human tonsil or spleen cell-derived immune organoids have been used to model productive HIV infection and test the effects of antiviral treatments and immune cell-based interventions. These co-culture systems provide a more comprehensive platform to dissect the crosstalk between pathogens, target tissues, and immune responses.

In addition to human organoids, animal-derived organoids are gaining attention for studying zoonotic infections and comparative virology. Bat lung organoids have been established to investigate bat-specific viral tolerance and susceptibility, offering insights into the natural reservoirs of viruses like SARS-CoV and Ebola (Elbadawy et al., 2025). Such models complement human organoids by enabling cross-species analyses that can inform pandemic preparedness and zoonotic disease control. Continued development of organoid biobanks and standardized protocols will enhance reproducibility and facilitate large-scale studies across different infectious agents and host backgrounds (Ya et al., 2024).

Taken together, infectious disease organoid models represent a significant technological leap in biomedical research. They provide versatile, physiologically relevant platforms that bridge the gap between simplistic *in vitro* systems and complex *in vivo* models, enabling more accurate studies of pathogen biology, host responses, and therapeutic interventions. Future efforts to improve organoid complexity, incorporate vasculature and immune components, and enhance maturation will further increase their utility in personalized infection modeling and drug discovery, ultimately improving outcomes for patients with infectious diseases.

## 5 Integration, industrialization, and future perspectives

### 5.1 Genome editing for therapeutic applications

The integration of organoid technology with genome editing has opened new avenues for precision therapy, enabling tailored interventions that address individual genetic and pathological profiles. Genetically modified organoids, particularly those derived from patient-specific induced pluripotent stem cells (iPSCs), serve as physiologically relevant platforms for developing targeted treatments. Using advanced CRISPR-Cas systems (knock-out and knock-in), researchers can precisely modify disease-associated genes within organoids, generating isogenic models that recapitulate disease phenotypes (Lee et al., 2026; Ramakrishna et al., 2021). High-efficiency CRISPR delivery methods, such as ribonucleoprotein (RNP)-mediated delivery and nanoblade technology, have significantly improved editing outcomes in organoids (Cao et al., 2026; Tiroille et al., 2023), enabling rapid generation of genetically defined models for therapeutic validation.

Beyond cancer modeling, organoid-based genome editing has shown promise for correcting monogenic disorders. Allele-specific CRISPR/Cas9 has been used to correct dominant mutations in the CRX gene in retinal organoids, achieving partial phenotypic rescue for inherited retinal dystrophies (Chirco et al., 2021). Prime editing has successfully corrected CFTR mutations in patient-derived intestinal organoids, restoring functional protein expression with high precision and minimal off-target activity (Bulcaen et al., 2024; Geurts et al., 2021). Mitochondrial base editing in liver organoids has enabled functional correction of mitochondrial DNA mutations linked to metabolic disorders (Joore et al., 2025). These platforms serve as preclinical models for evaluating gene correction strategies tailored to individual patients.

The combination of organoids with immune and cell therapies represents another frontier. Tumor organoids co-cultured with autologous immune cells (e.g., T cells) enable *in vitro* reconstruction of patient-specific tumor-immune microenvironments, allowing evaluation of immunotherapies and optimization of treatment regimens (Jeo et al., 2023; Zhou et al., 2024; Ning et al., 2024). Genome editing has also been used to generate immune-evasive pluripotent stem cell lines (Zahedi, 2026). In regenerative medicine, gene-edited iPSC-derived organoids are being developed for transplantation ADDIN EN.CITE (Turhan et al., 2021; Novelli et al., 2022). The convergence of organoid technology, genome editing, and immunotherapy paves the way for highly personalized treatment modalities. These applications currently reside at Technology Readiness Level (TRL) 4–5, with gene correction strategies validated in preclinical organoid models but not yet in clinical trials.

### 5.2 Automation, bioprinting, and organoids-on-chip

Commercial and technological advances have driven the industrialization of organoid platforms. Automated high-throughput culture and analysis systems, such as organo-on-pillar (high-TOP) platforms and pillar plate-based spheroid transfer methods, enable standardized, reproducible, and scalable organoid production (Jun et al., 2023). These innovations address previous bottlenecks related to variability and low throughput, facilitating integration into pharmaceutical screening pipelines and clinical decision support.

Organoids-on-a-chip, which combine organoids with microfluidic organ-on-chip technologies, enhance physiological relevance by mimicking dynamic tissue microenvironments. This improves predictive accuracy for drug responses and disease modeling (Liu K. et al., 2025; Wang H. et al., 2024). Bioprinting technologies allow precise spatial control over cell placement and organoid architecture, reducing batch-to-batch variability and enabling scalable production suitable for high-throughput drug screening (Boerstle et al., 2025; Hu et al., 2025). These engineering approaches provide robust, scalable, and physiologically faithful models, accelerating industrial translation. These platforms are currently at TRL 5–6, with pilot integration into pharmaceutical screening pipelines and ongoing validation for clinical decision support.

### 5.3 Regulatory, ethical, and commercialization considerations

Widespread clinical application of organoid technology requires addressing regulatory and ethical challenges. The absence of universally accepted standards for organoid manufacturing, quality control, and functional evaluation hampers regulatory approval and commercialization. Initiatives such as the Organoid Standards Initiative have been established to develop comprehensive guidelines covering material selection, production procedures, and endpoint quality assessments for various organoid types (e.g., liver, intestine, heart) (Ahn et al., 2024).

Animal-free extracellular matrix hydrogels are being explored to support animal-free *in vitro* models (Zhou L. et al., 2025; Nitsche et al., 2025). Ethical concerns related to donor consent, data privacy, and potential clinical use of organoids necessitate transparent policies and multidisciplinary collaboration among academia, industry, and regulatory bodies. Integration of artificial intelligence in organoid research holds promise to enhance data analysis and clinical evaluation (Ma W. et al., 2025).

A critical gap in the clinical translation of organoid technology is the absence of harmonized Good Manufacturing Practice (GMP) guidelines specific to organoid production. GMP-compliant organoid manufacturing requires defined, xeno-free culture matrices (replacing tumor-derived Matrigel), clinical-grade recombinant growth factors, and closed-system bioreactors to minimize contamination risk. Several groups have begun developing GMP-compatible protocols, but no consensus standard has been established across regulatory jurisdictions.

From a regulatory perspective, both the FDA and the European Medicines Agency (EMA) classify patient-derived organoid-based drug sensitivity tests as *in vitro* companion diagnostic devices or laboratory-developed tests (LDTs), subject to premarket review under applicable medical device regulations. The FDA’s framework requires analytical validation (demonstrating accuracy, precision, and reproducibility), clinical validation (demonstrating correlation with patient outcomes), and clinical utility (demonstrating improved patient outcomes) before organoid-based assays can guide treatment decisions. The EMA’s approach under the EU IVDR (*In Vitro* Diagnostic Regulation) similarly requires evidence of clinical performance and risk-based conformity assessment. To date, no organoid-based drug sensitivity assay has received FDA premarket approval or CE marking under IVDR, underscoring the early stage of regulatory readiness.

Validation requirements for organoid-based clinical decision-making should include: (1) prospective clinical trials demonstrating that organoid-guided treatment selection improves progression-free or overall survival compared to standard care; (2) standardized operating procedures for tissue acquisition, organoid culture, drug testing, and result reporting; (3) external quality assessment schemes to ensure inter-laboratory reproducibility; and (4) qualified positive and negative reference controls. Without these validation frameworks, organoid drug sensitivity results risk being misinterpreted or over-interpreted by clinicians, potentially leading to suboptimal treatment decisions.

Clinical trials increasingly employ organoids as predictive avatars for personalized medicine, particularly in oncology. Patient-derived organoids enable *ex vivo* drug sensitivity testing that informs individualized treatment regimens ADDIN EN.CITE (Le Compte et al., 2023; Li et al., 2024). However, clinical adoption remains limited by batch-to-batch variability, lack of standardized protocols, and the complexity of fully recapitulating tissue microenvironments. Current clinical implementation of organoid-guided drug testing is at TRL 6–7, with prospective clinical trials underway but no regulatory approval yet granted for routine clinical use.

### 5.4 Remaining challenges and future roadmap

**FIGURE 4:**
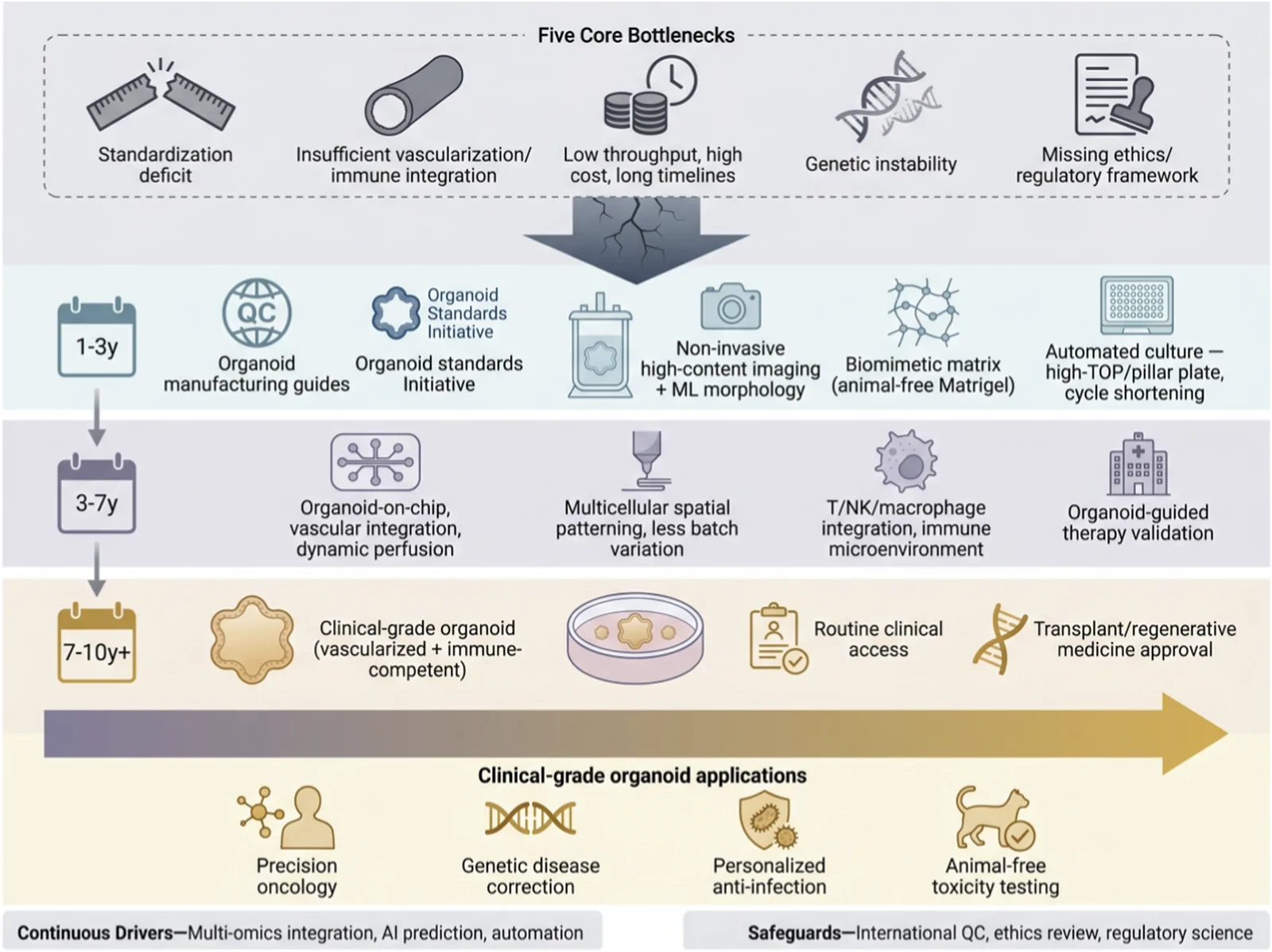
From current bottlenecks to clinical vision: the organoid technology development roadmap. This figure was created with the assistance of AI-based illustration tools and subsequently reviewed and edited by the authors for scientific accuracy.

Despite significant progress, several core bottlenecks must be overcome before organoid technology can achieve its full clinical potential Figure 4. At present, clinical implementation remains largely at the research stage (TRL 3–4), with only a few pilot clinical studies exploring organoid-guided treatment selection, and no broadly validated regulatory pathways have been established. Key challenges include the lack of standardization, which leads to inter laboratory variability in culture protocols, matrices, and media, thereby limiting reproducibility; the absence of functional vasculature and complete immune niches in current organoids, which restricts their ability to model systemic drug distribution and immunotherapy responses; the high cost and prolonged turnaround time of organoid establishment and drug testing, often incompatible with urgent clinical decision-making; difficulties in scalable, high-throughput production of quality-controlled organoids; and the lack of validated quality metrics and manufacturing standards, which delays regulatory acceptance. To address these issues, the proposed roadmap envisions: (1) short [TRL 3–5]-term development of internationally standardized quality control metrics and AI-assisted real-time monitoring; (2) medium [TRL 5–7]-term integration of vascular networks and immune components via microfluidic perfusion and bioprinting, coupled with multi-center clinical studies validating organoid-guided treatments; and (3) long [TRL 7–9]-term achievement of fully vascularized, immune-competent organoids that model systemic drug distribution, alongside regulatory approval for routine clinical use. By systematically tackling these challenges, organoid technology can be translated from bench to bedside, ultimately enabling personalized diagnostics and therapies.

## 6 Conclusion

Organoid technology has fundamentally reshaped personalized medicine by enabling patient-specific, three-dimensional models that faithfully preserve tissue architecture, cellular heterogeneity, and disease phenotypes. In this review, we have systematically charted the evolution of organoids from basic culture systems toward clinical translation, highlighting their expanding roles in oncology, genetic disorders, infectious diseases, and drug development, while critically discussing remaining bottlenecks such as standardization, vascularization, immune integration, and scalability. In summary, organoid technology stands at the cusp of clinical application, and with sustained interdisciplinary collaboration and targeted innovation, it is poised to revolutionize precision medicine by delivering truly individualized diagnostics and therapies.
